# Practical Observations on Various Subjects Relating to Midwifery

**Published:** 1837-07

**Authors:** 


### Art. XIV.

1. *Practical Observations on various Subjects relating to Midwifery.* By James Hamilton, m.d. f.r.s.e., Professor of Midwifery, &c. Parts I. and II.—*Edinburgh,* 1836.
2. *A Letter addressed to* Dr. Forbes *and* Dr. Conolly, *Editors of the British and Foreign Medical Review.* By Dr. Hamilton, Professor of Midwifery, &c.—*Edinburgh,* 1837.—8vo. pp. 20.

Our readers will recollect that we had occasion to notice the first Part of Dr. Hamilton's work in a former Number. The second Part is now before us, as well as the long Letter, in the form of a pamphlet, which Dr. Hamilton has done the Editors of this Journal the honour of addressing to them. It was our intention to have given our readers an account of Dr. H.'s new volume in the present Number; but, as we have been unable to find room for the article which we had prepared on it, we must content ourselves with briefly adverting in this place to the *Reclamation* which the author has thought it necessary to put forth in reference to certain of our comments on his former volume. We are induced to do so solely because the matters in discussion are of an important practical nature; as we altogether disclaim the purpose of entering, at any time, or with any person, upon questions of mere controversy, originating in difference of opinion between ourselves and the authors of the works reviewed in this Journal.

We refer to our pages for the proof that we belong not to the family of Dr. Johnson's critic in the Rambler, who "knits his brow, and raises his voice, and rejoices whenever he perceives any tokens of pain excited by the pressure of his assertions or the point of his sarcasms." We have but the one object in view for which Dr. Hamilton gives us credit,—by candid and liberal criticism to promote the improvement of science, and not to depreciate the character of those who exercise it. It is true our criticisms are anonymous, but they are couched in precisely the same terms, and influenced by precisely the same spirit, as if our names were given to the world. We must, however, claim the privilege of freedom of discussion, and we cannot condescend to the servility of flattery. We are sincerely sorry to find that any comments of ours have given uneasiness to Dr. Hamilton; for, in common with, we doubt not, the whole of his brethren, we have a very high respect for the devotion with which he has successfully dedicated a long life to the improvement of his profession; and we confess that, in commenting upon his works, we are afraid of not giving him credit for all the originality that fairly belongs to him. His peculiar views and practical doctrines have been so widely disseminated by the numerous pupils he has for so many years instructed, that, in all probability, opinions and improvements, which he first suggested, are now often met with in the writings of others, where, if they are not specially claimed as the property of the author, they are not, and perhaps could not, be traced to the source from whence they were at first derived.

The practical subjects upon which we have differed from Dr. Hamilton in our notice of his work in No. V. of our Journal, and which he brings under our review in the hope that, from our candour, we shall see reason to change our opinions, are the following:

Firstly, in cases of prolapsus uteri, Dr. Hamilton states that pessaries "can only act as palliatives, whatever may be the degree of the disease." We answer, that pessaries, properly used, may, and sometimes do, *cure* the prolapsus. We know this from our own experience. We cannot admit that "they *necessarily* keep up a constant irritation in the passage." We have frequently applied them, and the patients have worn them for a considerable time with the greatest comfort and relief, and without the slightest uneasy sensation being produced by them. It is true that, "unless they are properly adapted, they make injurious pressure on the contents of the pelvis." But this is merely an objection to the abuse of the instrument. Dr. H.'s fourth objection is, that, if the pessary be "not frequently taken out and cleaned, it becomes encrusted with a calcareous matter, which proves highly irritating." Granted; but every practitioner guards against this mischief by giving proper instructions to his patient. In severe degrees of prolapsus uteri, whatever may be the treatment adopted, the patient may long, and perhaps for life, require medical care; but we know, from cases which we have treated, that there are very many exceptions to the alleged fact, that pessaries "subject the patient to the charge of the medical attendant for life." We have not and do not deny that "cases from time to time occur where no ordinary pessary can be retained." We must doubt the propriety of "banishing" any mode of practice, because in *one* case it had been grossly abused by the carelessness of the patient. In the case related by Dr. H., at page 26, "minute directions were given to have the pessary withdrawn once a week, and carefully cleaned." The patient, however, never withdrew the instrument: it became impacted, and made its way into the rectum. "From the date of that case, the author has never sanctioned the use of pessaries!" We have not, in our review, objected to the treatment advised by Dr. Hamilton; but we see no reason to retract our opinion that both he and Osiander have been too hasty and too exclusive in their condemnation of an instrument, which we are often compelled to use, and which, when skilfully employed, is certainly not liable to produce the mischievous effects which they attribute to it. We repeat our doubts as to the propriety or safety of recommending a patient with prolapsus of the uterus to have recourse to "walking exercise," however "cautiously begun, whatever be the feelings of the patient," (p. 30;) but, upon the authority of Dr. Hamilton, we have expressed our determination to give this novel practice "a fair trial;" and so we shall.

Secondly, our "observations on polypous excrescences do surprise" Dr. Hamilton. "How there could be any difference of opinion on the diagnostic marks is to me unintelligible," says Dr. H. We refer to Dr. Gooch's* cases, and to Mr. Arnott's Lecture.† We need not accumulate evidence upon a practical point that is so perfectly intelligible. All that we have said in our review, however, is, that "prolapsus can only be distinguished by actual examination; and, even with this assistance, the young practitioner may be mistaken in his diagnosis." So far from retracting this opinion, we add that the difficulty of diagnosis may extend to the old and experienced. One case in point we have quoted from Velpeau. Gooch says that, in the treatment of polypus, "the chief difficulty is in the diagnosis;" and that, "when a tumour is detected, he has known the most experienced practitioners hesitate about its nature."‡ We cannot modify any part of the opinion we have stated in our review concerning the "*general*" superiority of excision to the use of the ligature in cases of polypus; and we must be allowed to repeat, that Dr. Hamilton is *quite in error* as to "British practitioners" being "universally agreed that the safe mode of operating is by ligature," or that "French surgeons have lately preferred the double operation of tying the polypus, and then cutting it off." We adhere strictly to the assertions we have made in our review, that excision is now *generally* preferred here and in France. Dr. Hamilton states it as his "opinion" that excision, even if practicable, is a most hazardous operation, unless where the excrescence does not exceed the size of a filbert." He must allow us to reply, that an "opinion," by whomsoever expressed, cannot be opposed to the result of experience. Sir Benjamin Brodie permits us to state that he has several times removed large polypi by excision, and that no danger ever arose from the practice. We saw Mr. Arnott remove a polypus, which completely blocked up the vagina, and which was nearly as large as a double fist. "Not a couple of ounces of blood followed the operation,"§ and in less than a month the patient was well.

* Account of some of the most important Diseases peculiar to Women, p. 261, &c.
† Med. Gazette, vol. xviii. p. 410.
‡ Loc. cit. 261; and Arnott's Case, loc. cit. § Loc. cit. p. 413.

In several cases we have ourselves adopted the same practice, and with the same success. Dupuytren operated by excision in two hundred cases, and *only twice* met with hemorrhage to any extent; and in both cases it was quickly stopped by plugging the vagina. Again, we refer to the best authorities upon the subject to prove that Dr. Hamilton is mistaken in asserting that "the *only* danger attending the ligature arises from the risk of including a portion of the uterus." Irritation, fever, and death have resulted from the practice when the ligature was applied to the stalk of the polypus; and the fatal cases, as related by Dr. Hamilton, at p. 59 *et seq.* of his work, would, in our opinion, lead most practitioners to doubt the safety of the practice, even where no portion of the uterus was involved in the ligature. We confess we know nothing of the advantages of silver wire over other kinds of ligature. Lecat, we believe, first recommended it; it is objected to by Monfalcon,* Burns,† &c.

Lastly, as to the evidences or signs of human pregnancy, a subject, in many points of view, of the very utmost importance, and concerning which it is essentially necessary we should not assume a degree of certainty we do not possess. Dr. Hamilton does "not venture to express what he thinks" on our remarks upon his observations on the evidences of pregnancy. And "by what mode of reasoning any individual acquainted with the economy of the gravid uterus could suppose it possible for menstruation to continue during pregnancy," appears to Dr. H., "considering the present state of our knowledge of anatomy and physiology, to be very inexplicable." If Dr. Hamilton will do us the favour to refer to Dewees,‡ Desormeaux,§ Mayo,|| Velpeau,¶ or, still better, to Dr. Montgomery,** whose *candid* and elaborate consideration of the signs of pregnancy deserves the highest praise, he will find the mode of reasoning that has been adopted, and the facts that have been recorded in proof of the opinion which we, in common with the great majority of practitioners and writers in midwifery maintain, that the menses *may* flow during pregnancy: or, if we must guard against a quibble, that such a periodical discharge may appear that cannot be distinguished from the menses.

The confidence, too, with which Dr. Hamilton regards the areola as an *invariable* sign of pregnancy, is opposed to the opinions of most practical observers and writers; and we must be allowed to add, that the cautious statements of Denman, Gooch, &c. are much too cavalierly treated and dismissed. How can Dr. H. take upon himself, with propriety, to declare that the assertion of Denman, of the areola being formed in many of the complaints which resemble pregnancy, is quite inconsistent with the observation of *every* modern practitioner? We have for many years paid great attention to this subject, and we believe we have seen, to use no stronger term, a completely formed areola in cases of dysmenorrhœa. Dr. Hugh Ley was of the same opinion. Nobody, we believe, doubts the *great* value of these signs of pregnancy, nor the *strong* presumptive evidence they afford; but we object to their being adduced as *invariable* proofs of pregnancy in the early months, as strongly as we do to the belief of Dr. Hamilton, that, in the latter months, the movements of the

* Dict. des Sciences Médicales, t. xlix. p. 248.

† Midwifery, eighth Edition, p. 118.

‡ Midwifery, p. 93 et seq.

§ Dict. de Médicine, t. xiv. p. 185.

|| Physiology, third Edition, p. 371.

¶ Accouchemens, t. i. p. 182.

** Cyclopædia of Pract. Med. vol. iii. p. 471.

infant can *always* be distinguished by an attentive practitioner. Dr. H. admits that "cases are recorded, upon apparently good authority too, where it was supposed that, although pregnancy proceeded safely to the full period, the movements of the infant, though alive, had never been perceived by the parent, and could not be detected by the practitioner." Such is our own, and such is the general conviction of the profession; and, again, we must object to the dogmatic manner in which Dr. Hamilton gets rid of the difficulty, by saying "that he holds all those alleged cases to be the offspring of prejudice and credulity." Upon this, and upon all the other signs of pregnancy which Dr. Hamilton would almost alone have us look upon as "invariable," we might have adduced, in opposition to him, a host of authorities of the highest reputation, who rely upon undoubted facts for the support of their opinions.

We have now noticed all the important points of Dr. Hamilton's letter, and we leave our readers to judge whether we have or have not substantiated the remarks we made in our review.

---

